# Assessment of cognitive function in long-term Hodgkin lymphoma survivors, results based on data from a major treatment center in Hungary

**DOI:** 10.1007/s00520-022-06918-6

**Published:** 2022-03-11

**Authors:** Ferenc Magyari, István Virga, Zsófia Simon, Zsófia Miltényi, Anna Illés, Karolina Kósa, Tibor Ivánka, Roland Berecz, Anikó Égerházi, Árpád Illés

**Affiliations:** 1 Division of Haematology, Department of Internal Medicine, University of Debrecen, Debrecen, Hungary; 2 Doctoral School of Clinical Medicine, University of Debrecen, Debrecen, Hungary; 3 Department of Anesthesiology and Intensive Care, University of Debrecen, Debrecen, Hungary; 4 Department of Behavioral Sciences, University of Debrecen, Debrecen, Hungary; 5 Department of Psychiatry, Faculty of Medicine, University of Debrecen, Debrecen, Hungary

**Keywords:** Hodgkin lymphoma, Cognitive impairment, Computerized neuropsychological test battery (CANTAB), Health-related quality of life, Employment status

## Abstract

**Introduction:**

Nowadays, more than 80% of newly diagnosed classical Hodgkin lymphoma (HL) patients can be cured and become long-term survivors due to risk and response-adapted treatment strategies. A well-known side effect is cognitive dysfunction that appears in HL patients after chemotherapy. In the present study, we aimed to measure cognitive dysfunction in our HL patients in this study and to find potential correlations between patient-related factors, the signs and symptoms of their diseases, or therapeutic factors.

**Methods:**

We carried out a computer-assisted assessment (CANTAB) of cognitive dysfunction in 118 patients. We examined the domains of visual memory, attention, working memory, and planning.

**Results:**

The median age of 64 females and 54 males at diagnosis was 29 (13–74) and 41 (21–81) years at the completion of CANTAB. Fifty-two percent of all patients showed cognitive impairment. Attention was impaired in 35% of patients, the working memory and planning were impaired in 25%, while visual memory was affected in 22%. All the three functions showed a significant association with inactive employments status. A close correlation was found between visual memory/working memory and planning, higher age at HL diagnosis or the completion of CANTAB test, and disability pensioner status.

**Discussion:**

Our investigation suggests that patients with inactive employment status and older age require enhanced attention. Their cognitive function and quality of life can be improved if they return to work or, if it is not possible, they receive a cognitive training.

**Supplementary Information:**

The online version contains supplementary material available at 10.1007/s00520-022-06918-6.

## Introduction

Hodgkin lymphoma (HL) primarily affects active young adults: a significant portion of patients were working-age young adults at the time of diagnosis. Therefore, the social and economic implications of the disease outweigh its incidence rate. In 2020, 83087 new HL patients were diagnosed worldwide, and thus HL accounted for 0.4% of total cancer cases [1]. The incidence rate is 2–3/100,000 inhabitants in Hungary, which means approximately 2–300 newly diagnosed cases each year [2, 3]. Due to up-to-date methods of clinical investigation and risk- and response-adapted therapy, more than 80% of patients with HL show long-term survival and recovery. Parallel to this, treatment-related long-term complications have come to the fore. The long-term survival rate is mainly decreased by developing second malignancies and the appearance of organ damage (heart, lungs, thyroid gland, etc.). Nowadays, the quality of survivorship is becoming more prominent. Therefore, treatment-related side effects must be focused on, but health-related quality of life and patients’ return to work after successful treatment should also be considered [4]. Based on literature data, cancer-related cognitive impairment (CRCI) is a common adverse effect experienced by patients during and after chemotherapy (chemobrain) for non-central nervous system cancer/lymphoma [5]. Many factors influence CRCI. Treatment-related factors include the type of chemotherapy and its direct and indirect neurotoxicity caused by these, which can occur even if the blood-brain barrier is intact. Non-chemotherapy-related factors include age, education, depression, and psychological stress. In addition, the type of disease must also be considered, as not all hematological disorders require immediate treatment (indolent malignancies) [6–8]. CRCI mainly affects the functions of the domains of attention, working memory, and planning [7, 9, 10]. There are few published data available on cured HL patients [6, 11, 12]. Our study aimed to measure cognitive dysfunction in our regularly followed-up HL patients, finding potential correlations between patient-related factors, specific signs and symptoms of their diseases, and therapeutic factors.

## Methods

### Subjects and data collection

A cross-sectional survey was conducted at the Division of Hematology of the University of Debrecen. We designed the study for approximately 100 of the 301 patients who had been regularly followed up. Exclusion criteria were lack of signed informed consent, age under 18 years at the time of the survey, and the presence of central and peripheral nervous system involvement with HL. Altogether, 118 adult HL survivors were identified between June 1, 2012, and January 31, 2016, at our outpatient clinic diagnosed with HL between January 1, 1969, and July 1, 2013. Age, gender distribution, clinical stage, and type of treatment of the selected patients were no different from that of our population of Hodgkin lymphoma patients. Based on the hospital records of the patients, diseases diagnosed before the initiation of HL treatment were defined as comorbidities, whereas treatment-related side effects were defined as conditions diagnosed in the follow-up phase after HL treatment.

### Treatment protocols

Primary chemotherapy involved CV(O)PP (cyclophosphamide, vinblastine (vincristine), procarbazine, and prednisolone) before. After 1990, COPP/ABV (cyclophosphamide, vincristine, procarbazine, prednisolone/adriamycin, bleomycin, and vinblastine) was used. Since 1999, the ABVD (adriamycin, bleomycin, vinblastine, and dacarbazine) protocol has been most frequently applied. In cases of relapse, DHAP (dexamethasone, cytarabine, and cisplatin), ICE (ifosfamide, carboplatin, etoposide), or IGEV (ifosfamide, gemcitabine, vinorelbine) regimens and autologous hemopoetic stem cell transplant are included. Radiotherapy of the involved or extended field, mantle, and inversed Y or (sub)total nodal type was administered by a telecobalt device before, 2000, and by a linear accelerator afterwards. Extended and involved-field radiotherapy was used before (mean dose, 40 Gy) and after 1998 (mean dose, 33 Gy), respectively (Supplementary Table 1).

### Study design and assessment

Subjects were asked to perform a series of 13 computerized neuropsychological tests of the Cambridge Neuropsychological Test Automated Battery (CANTAB, Cambridge Cognition, Cambridge, UK). CANTAB has been used and proved to be a useful tool to assess cognitive functions in various neurological and psychiatric disorders [13, 14]. Subjects were seated at a comfortable height, approximately 0.5 m from the monitor, and were instructed to carry out the tasks by touching the screen. After an initial explanation and completing a simple “motor screening task” successfully (touching the center point of flashing crosses on the screen), subjects were given the following tests in the following order (the technical description of the tests can be found on the Cambridge Cognition’s website: http://www.cantab.com):

- Big Little Circle (BLC): a two-stimuli visual discrimination and category achievement test. Spatial working memory (SWM): this task assesses the subject’s ability to retain spatial information and manipulate remembered items in working memory.
- Reaction time (RTI): The task is designed to measure the subject’s speed of response to a visual target where the stimulus is either predictable (simple reaction time) or unpredictable (choice reaction time). A manual switch is used for the task.
- Spatial span (SSP): A computerized version of the Corsi blocks, a test of span for spatial items similar to “digit span” tests for verbal items.
- Pattern recognition memory (PRM): An examination of visual recognition memory in a 2-choice forced discrimination paradigm.
- Spatial recognition memory (SRM): This task tests visual-spatial memory in a 2-choice forced discrimination paradigm.
- Paired associate learning (PAL): Assessment of simple visual pattern and visuospatial associative learning, which contains aspects of a delayed response procedure and a conditional learning task. Successful performance in the PAL test requires both the elaboration of “frontal strategies” and the “mnemonic processes” of the medial temporal lobe. They should pay attention to both the stimuli and their spatial position.
- Intra/extradimensional shift task (IED): A test of rule acquisition and reversal, featuring visual discrimination and attentional set-shifting and analogous to a category change in the Wisconsin Card Sorting Test.
- Match to sample visual search (MTS): A two-stimuli visual discrimination and category achievement test.
- Delayed matching to sample (DMS): This task tests visual memory in a 4-choice delayed recognition memory paradigm.
- Stockings of Cambridge (SOC): The task is analogous to the “Tower of London” test and assesses the subject’s ability to engage in spatial problem-solving. This test makes substantial demands on executive function.
- Rapid visual information processing (RVP): It is a visual continuous performance task, using digits rather than letters. Results were compared to the internal normative database of CANTAB, involving 3,000 healthy volunteers, and were matched for age groups and gender. CANTAB tests were previously validated among healthy Hungarian volunteers showing no statistically significant differences in the cognitive performance compared to the internal normative database [13, 15].

We examined the domains of visual memory (PAL, DMS, SRM), functions of attention (IED, RTI, RVP, PRM), working memory, and planning (SSP, SWM, SOC). Although the CANTAB battery emphasizes assessment of frontostriatal functions (SWM, IED, SOC, and SWP), it also includes tests sensitive to temporal lobe (PAL, DMS, PRM, SSP) function [16].

### Questionnaires

HL survivors were asked to complete a standardized, self-administered, and validated Hungarian questionnaire, which included items on socio-demographic status (place of residence, marital status, educational level, employment, important life events after lymphoma treatment) and psychiatric treatment (date and type of medication) at the time of diagnosis, as well as the scales listed below. Data on the disease and its treatment were based on hospital records.

The Hungarian version of the Hospital Anxiety and Depression Scale (HADS-14) has been used in studies of distress among cancer patients in general. Each of the 14 items is scored on a 4-point scale (0–3). Sum scores for the anxiety and depression subscales are calculated by simple addition. The constructors of HADS recommended two possible cut-offs (8 or higher or 11 or higher on either scale) for case definition. In this study, caseness refers to the lower cut-off [17, 18].

The General Health Questionnaire (GHQ-12) is the most extensively used screening instrument for common mental disorders, in addition to being a general measure of psychiatric well-being. Each item is scored on a 4-point scale (corresponding to a symptom present: “not at all,” “same as usual,” “rather more than usual,” or “much more than usual”). It can be scored in a bimodal fashion (0–0–1–1), when final scores range from 0 to 12. According to this method, patients scoring five or more are considered: at risk of anxiety/depression [19].

The validated Hungarian version of the abbreviated Sense of Coherence (SOC-13) scale was used in the present survey to measure the overall capacity to cope with stressful situations. All 13 items are answerable on a Likert scale from 1 to 7, and total scores vary between 13 and 91. A higher score indicates a stronger SOC [19].

The Perceived Stress Scale (PSS) is the most widely used psychological instrument for measuring stress perception. Scores for the 4-item form range from 0 to 16. Potential responses range from 0 (never) to 4 (very often), and positively stated items are reverse coded before items are summed up with higher scores indicating more perceived stress [20].

The Dysfunctional Attitude Scale form A (DAS-A) is designed to measure the presence and intensity of dysfunctional attitudes. The higher the score, the more dysfunctional attitudes are characteristic of an individual. The 17 items are divided into two subscales: perfectionism and dependency. Each item is scored on a Likert scale from 1 to 7. Sum scores for either subscale are calculated by simple addition [21]. All questionnaires employed in the survey have been validated and extensively used in the international literature [22–25].

### Statistical analysis

Statistical analysis was performed using IBM SPSS 26 software. Data are described by the mean, standard deviation frequencies, and percentages. Categorical variables were compared between groups using chi-squared or Fisher’s exact test, as appropriate. Continuous variables were evaluated by independent samples t-test, Mann-Whitney test, and ANOVA or Kruskal-Wallis test. Spearman’s correlations were used measuring the relationship between two variables. Multiple/binary logistic regression (enter and forward likelihood ratio methods) was performed among HL survivors to identify predictors of cognitive dysfunctions. Odds ratios (OR) with 95% confidence intervals (CI) were estimated for the logistic regression models. Significance level was set at *p*<0.05. Since no control group was available, the participants’ Z-scores of all CANTAB subtests were calculated from median scores based on the normative database of 3000 healthy volunteers. The index scores of the patients and those of the normative database were compared using a one-tailed non-parametric t-test. Statistical analysis was carried out using GraphPad Prism 4.00 for Windows software (GraphPad Software, San Diego, CA, USA, http://www.graphpad.com). We regarded as having reduced cognitive function patients who performed worse than the normal population by 1.5 standard deviations or if the results of at least one test were already positive [9].

## Results

### Patient characteristics

One hundred twenty patients were asked to participate in our study. The refusal rate was below 5%. A total of 118 adult HL survivors completed the survey (64 females, 54%). The median age of the survivors at the time of diagnosis was 29 years at the time of diagnosis (range 13–74) and 41 years when the neurocognitive survey was performed (range 21–81). After the diagnosis, this investigation took place 11 years (range 0.4–44). The baseline characteristics of the survivors are presented in Table 1.

**Table 1:** Baseline characteristics of HL survivors. *ECOG PS* Eastern Cooperative Oncology Group Performance Status, *CR* complete remission, *PR* partial remission, *SD* stable disease, *ABVD* adriamycin, bleomycin, vinblastine, and dacarbazine

	All patients (*n*=118)
Mean age at survey (years)	45.73±14.37
Mean age at diagnosis (years)	32.45±12.89
Time elapsed (years)	13.29±9.93
Observation period (years)	
0–4	28 (24%)
5–9	25 (21%)
10–14	18 (15%)
>14	47 (40%)
Male	54 (46%)
Female	64 (54%)
Residence	
Village	26 (22%)
City	92 (78%)
Education level	
High school or below	79 (67%)
College or above	38 (33%)
Active employment status	69 (67%)
Inactive employment status	34 (33%)
HL stage	
I	5 (4%)
II	56 (47%)
III	42 (36%)
IV	15 (13%)
Bulk disease	21 (35%)
Without bulk	39 (65%)
B symptom	22 (37%)
No B symptom	38 (63%)
ECOG	
0	23 (22%)
1	31 (30%)
2	20 (20%)
3	21 (21%)
4	7 (7%)
Comorbidity	65 (55%)
No comorbidity	53 (45%)
Treatment	
ABVD	74 (64%)
Other	43 (36%)
Irradiation	93 (79%)
No irradiation	25 (21%)
Primary treatment response	
CR	112 (95%)
PR	4
SD	2
Treatment-related side effect	
Yes	65 (55%)
No	53 (45%)
Relapse	20 (17%)
No relapse	98 (83%)

### The measurement of neurocognitive function

**Fig. 1:**
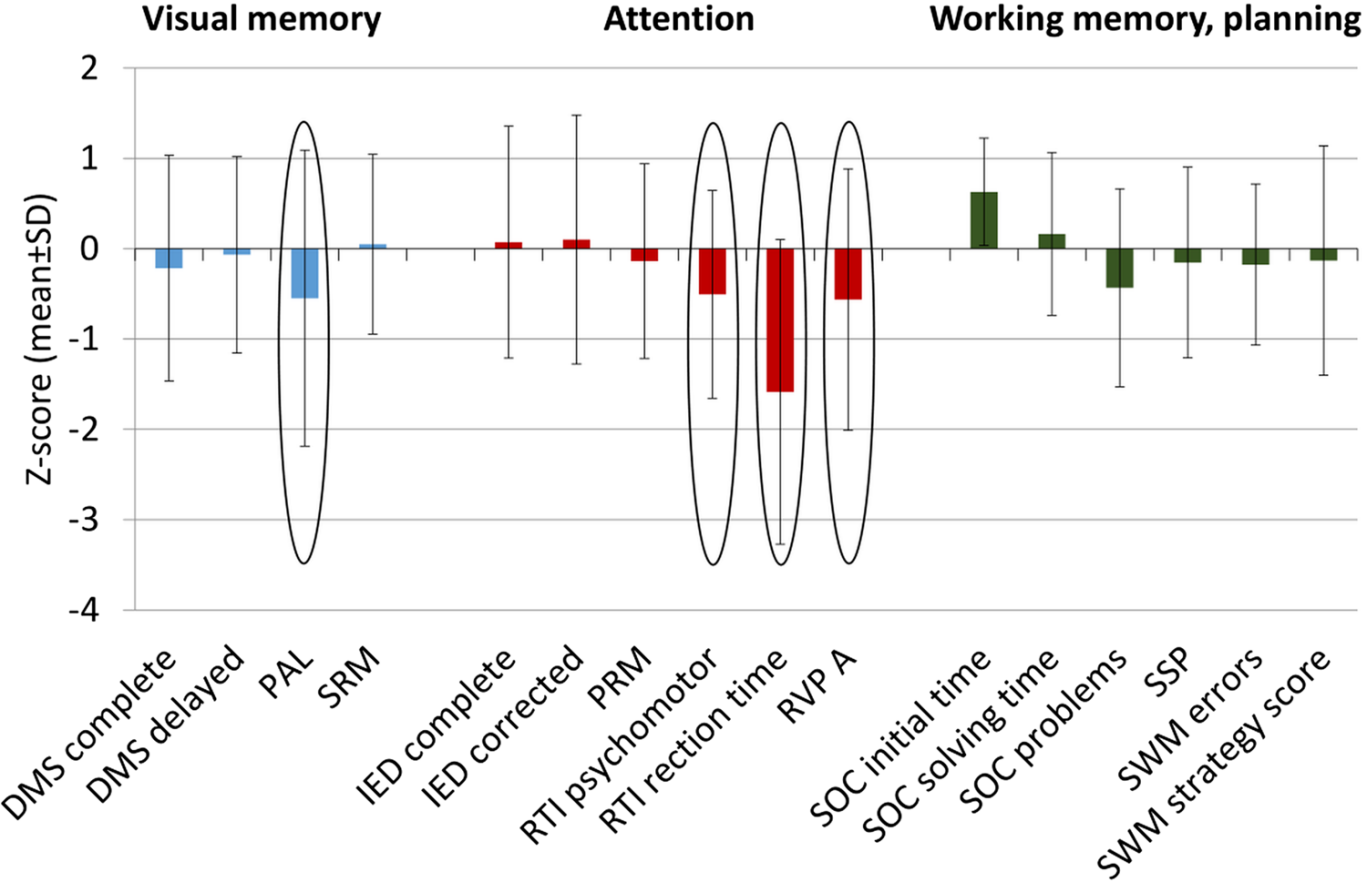
Difference between HL survivors (*n*=118) and the control CANTAB volunteers. DMS, simultaneous and delayed matching; PAL, paired association learning; SRM, spatial recognition memory; IED, intra/extra dimensional shift; PRM, pattern recognition memory; RTI, reaction time; RVP, rapid visual information processing; SOC, stocking of Cambridge; SSP, spatial span; SWM, spatial working memory

The pattern of median Z-scores of the CANTAB tests and their difference from the control scores measured in subjects with HL are presented in Figure 1.

As a next step, we investigated whether there are associations between positive neurocognitive subtest results (visual paired associate learning, reaction time and psychomotor speed, rapid visual processing) and patient-related factors, specific signs and symptoms of their diseases, or therapeutic factors. The PAL test was found significantly impaired in HL subjects at an older age at diagnosis and completion of the survey. The performance on RTI was prolonged in patients who were at an advanced stage with higher ECOG PS at diagnosis. A psychomotor RTI was strongly associated with the advanced stage at diagnosis. RVP test detected a significant impairment in patients at an older age at the time of HL diagnosis or when the survey was completed in village dwellers and patients with inactive employment status. These results are presented in Supplementary Table 2.

**Fig. 2:**
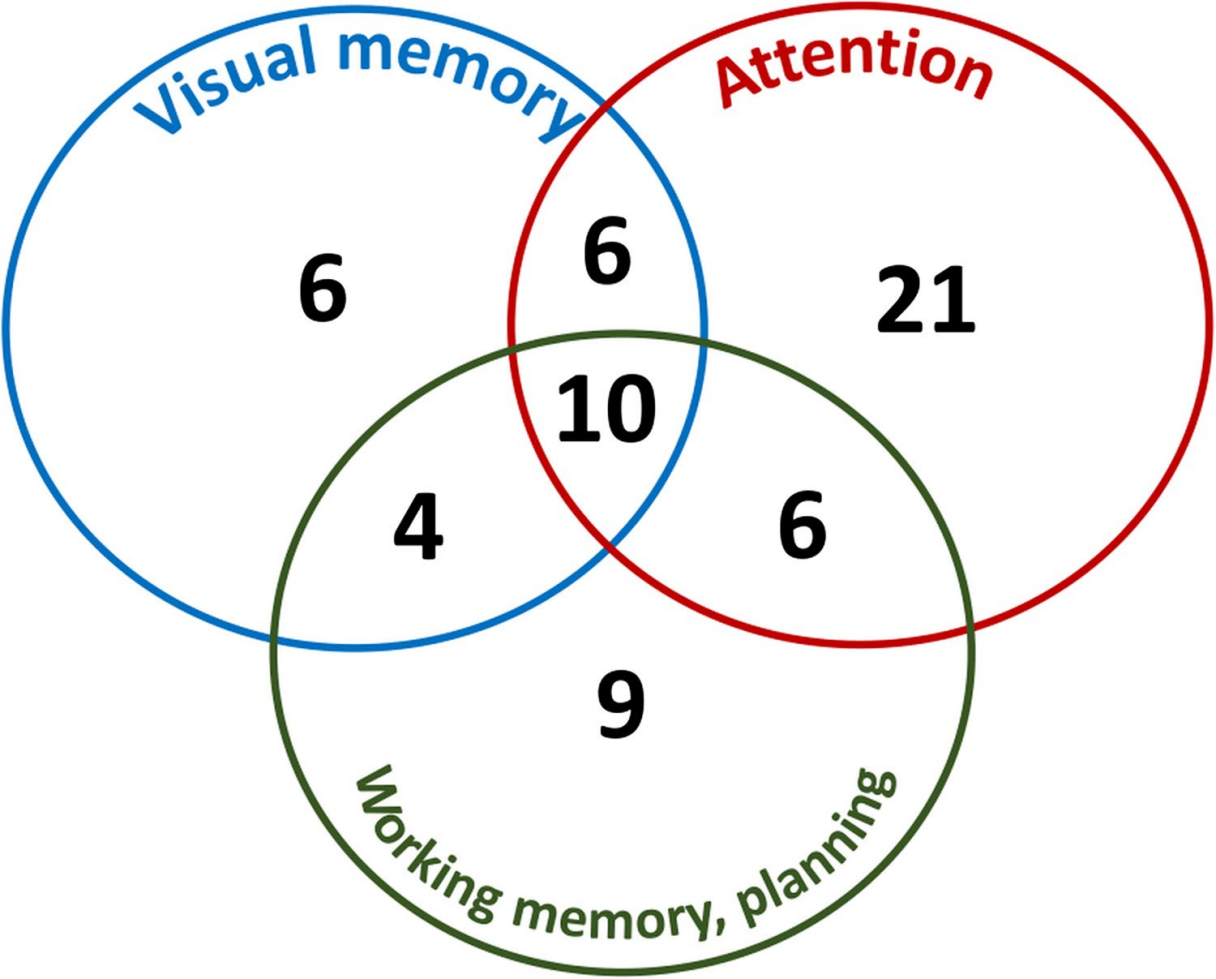
Impaired cognitive function (*n*=62) based on the visual memory, attention, and working memory/planning group

Fifty-two percent of all patients (*n*=62) showed cognitive impairment based on at least one positive subtest. Attention was impaired in 35% (42/118) of patients. Working memory and planning were damaged in 25% (30/118), while the visual memory was affected in 22% (26/118 patients) (Figure 2). One domain was found positive in 36 patients (30%, 36/118), two domains in 16 patients (14%, 16/118), and three in 10 patients (8%, 10/118). We defined severe mental vulnerability as having abnormal cognitive functions in all the three domains. The ten most affected HL survivors were at an older age at HL diagnosis (>=30 years, *p*<0.001) or when the survey was completed (>=40 years, *p*<0.001), had disability pension status (*p*=0.044), or used central nervous system drug (*p*=0.0239) (data not shown).

All three cognitive domain’s functions showed significant correlations with inactive employment status. On the one hand, a close correlation was found between visual memory and age at diagnosis, the time of examination, and disability pension status. On the other hand, working memory and planning showed a close association with the age at diagnosis, the time of examination, and disability pension status (Table 2.).

**Table 2:** Predictive factors for visual memory, attention, and working memory/planning

	Impaired function	Normal function	*P*
**Visual memory**			
Age at CANTAB			0.002
>=40	21 (81%)	43 (47%)	
<40	5 (19%)	49 (53%)	
Age at HL dg.			0.001
>=30	20 (77%)	37 (40%)	
<30	6 (23%)	55 (60%)	
Disability pension	10 (40%)	16 (18%)	0.017
Other patients	15 (60%)	75 (82%)	
Employment status			0.034
Inactive	11 (52%)	23 (28%)	
Active	10 (48%)	59 (72%)	
**Attention**			
Employment status			0.049
Inactive	16 (46%)	18 (26%)	
Active	19 (54%)	50 (74%)	
**Working memory, planning**			
Age at CANTAB			0.045
>=40	21 (70%)	43 (49%)	
<40	9 (30%)	45 (51%)	
Age at HL dg.			0.001
>=30	22 (73%)	35 (40%)	
<30	8 (27%)	53 (60%)	
Disability pension	11 (38%)	15 (17%)	0.021
Other patients	18 (62%)	72 (83%)	
Employment status			0.015
Inactive	14 (52%)	20 (26%)	
Active	13 (48%)	56 (74%)	

### Independent predictive factors for domains of cognitive functions

Multivariate logistic regression analysis was carried out by starting with all potential determinant variables and eliminating the non-significant ones. Independent variables of visual memory and working memory/planning were age at diagnosis of HL and the age when the CANTAB survey was performed. The functions of all the three cognitive domains were associated with inactive employment status (Table 3).

**Table 3:** Odds ratios and 95% confidence intervals of cognitive domains

	ODDS	95% CI	*P*
**Visual memory**			
Age at CANTAB >=40 vs <40	4.786	1.662–13.784	0.004
Age at HL dg. >=30 vs. <30	4.955	1.817–13.509	0.002
Disability pension vs. other patients	3.125	1.190–8.204	0.021
Employment status Inactive vs. active	2.822	1.056–7.638	0.039
**Attention**			
Employment status In active vs. active	2.339	1.004–5.505	0.050
**Working memory, planning**			
Age at CANTAB >=40 vs. <40	2.442	1.007–5.921	0.048
Age at HL dg. >=30 vs. <30	4.164	1.668–10.396	0.002
Disability pension vs. other patients	2.933	1.153–7.463	0.024
Employment status Inactive vs. active	3.015	1.212–7.501	0.018

### Correlation between cognitive function tests and mental health questionnaires

The correlation between the cognitive and mental health tests is summarized in Table 4. Unfortunately, due to printing error, item 10 in the HADS questionnaire was missing 2 answer options (labeled 2 and 3). Twenty-eight percent of patients (*n*=33) gave a positive answer (greater than 0) to the question printed incorrectly. If the additional questions 2 and 3 had received a positive response, our results could have been further improved. Misprinting of the question could not have led to a false-positive result due to the nature of the questionnaire.

**Table 4:**
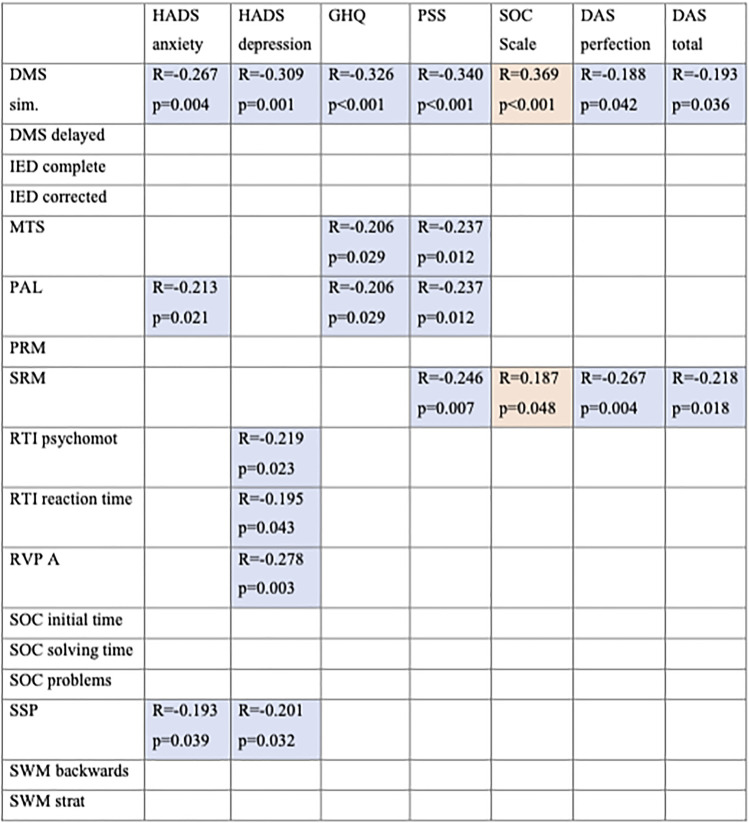
Significant correlations between cognitive subtest results and health-related quality of life (HRQOL) questionnaires. Spearman’s rank correlation coefficient was used to establish statistical significance

*DMS* simultaneous and delayed matching, *PAL* paired association learning, *SRM* spatial recognition memory, *IED* intra/extra dimensional shift, *PRM* pattern recognition memory, *RTI* reaction time, *RVP* rapid visual information processing, *SOC* stocking of Cambridge, *SSP* spatial span, *SWM* spatial working memory, *HADS* Hospital Anxiety and Depression Scale, *GHQ* General Health Questionnaire, *PSS* Perceived Stress Scale, *SOC* Sense of Coherence Scale, *DAS* Dysfunctional Attitude Scale. Blue colour indicates as a negative; orange colour indicates a positive correlation

## Discussion

Treatment of HL has become one of the success stories of oncohematology over the past decades. This has resulted in a continuously increasing HL survivor rates in patients at risk for long-term complications and health-related quality-of-life impairment. HL primarily affects active young adults: the median age of a significant portion of patients at the time of diagnosis was around 40 years. Therefore, the social and economic implications of the disease outweigh its incidence rate [26, 27]. Neuropsychological (NP) studies have shown cognitive dysfunction in 13–70% of patients receiving chemotherapy (solid cancers/hematological malignancies). However, 15–35% may experience permanent deficits well beyond the active treatment phase [28, 29]. CRCI is associated with a decrease in quality of life; reduced ability to work, read, or drive; and decreased social functioning. A significant proportion of clinical trials were performed among solid cancer patients (the most common type of breast cancer). However, most clinical trials in hematological disorders focus on survivors of acute lymphoblastic leukemia and primary central nervous system lymphoma [6, 30].33 There are few published data available on HL survivors [6, 11, 12, 31].

The present study results show that several cognitive domains are already impaired in long-term HL survivors. These data are consistent with the findings of previous studies [5, 11, 32, 33], made among survivors examined at least 5 years after completing chemotherapy. Based on the results of reaction time (RTI) and psychomotor speed subtests, there was a clear association with the advanced stage, which might emphasize the role and the cycles of chemotherapies among HL survivors. Our findings regarding reaction time are similar to preliminary data [12, 34], which had detected that receiving a lower number of chemotherapy cycles was associated with better neurocognitive performance. There are no literature data on ECOG PS in HL patients.

More than half of our long-term HL patients (52%, *n*=62) showed impairment based on at least one cognitive domain. Attention (and processing speed) was impaired in 35% of patients, working memory and planning (executive function) were damaged in 25%, while visual memory (memory and learning) was affected in 22%. A research group from Israel found impaired cognitive functions by CANTAB among their HL survivors (51 patients): executive functions were damaged in 41% of subjects, memory and learning in 28%, processing speed in 22%, and attention in 12%. Treatment completion of HL survivors was 6 months to 5 years [12]. Mariegaard et al. published that 39% of lymphoma survivors (115 patients, 65 with HL) showed impairment on executive function tests made with self-reported cognitive questionnaires (the mean period time elapsed after the completion of treatment was 29.6 months [31]. The remarkable differences between executive functions might be related to the period time elapsed from the treatment.

We identified associations between objective neurocognitive deficits (all three cognitive domains) and inactive employment status. Krull et al. reported favorable data on employment status and cognitive dysfunction in childhood HL survivors. Their analysis revealed that unemployed status (10 patients, compared with employed survivors) was related to significantly reduced motor speed, more severe impairments in working memory, and task efficiency [33]. Kiserud et al. reported that 102 unemployed people of 281 subjects with long-term survival after autologous hemopoietic stem cell transplant had poorer cognitive functions. Ehrhard et al. also confirmed that unemployed childhood non-Hodgkin lymphoma (NHL) survivors (187 patients) showed reduced processing speed, similar to our findings [35].

According to our results, being older at diagnosis or when completing the survey and disability pension status was related to poorer cognitive function. Preliminary data suggest that cancer and its treatment may compromise the normal aging process and increase cognitive impairment in those who are at the age of 65 years or older [11, 36]. However, the majority of HL survivors were in early and middle adulthood, so further research will have to justify additional factors which might lead to cognitive impairment.

We demonstrated significant reductions across a number of HRQOL domains in long-term HL survivors, which was related to neurocognitive impairment. The depression scale of HADS is correlated with reaction time assessments. At this time, we do not know that reduced cognitive function is a consequence or the cause of depression. The results published by Ehrhardt et al. suggest that objectively measured processing speed and survivor-reported executive function are associated with worse HRQOL among NHL survivors [37]. An Italian research group published that both subjectively and objectively assessed cognitive measures correlated negatively with most assessed HRQOL domains, indicating that the quality of life is worse when cognitive functioning is poor [38]. Based on previous results, impaired cognitive functions and poorer mental health might have a strong relationship. Recent data supporting a trend toward improved neurocognitive function after cognitive rehabilitation may provide means to find early detection and intervention strategies to improve HRQOL impairment among long-term lymphoma (HL) survivors [37, 39].

Our study is not without limitations. The main limitations of this research were its cross-sectional design, the absence of baseline neuropsychiatric (NP) tests, lack of matched healthy control population, and neuroimaging examination.

## Conclusions

According to literature data, NP tests to measure cognitive function often take more than 4 h and require trained investigators. CANTAB is a simple NP test, covering a wide range of cognition (visual memory, attention, work memory, and planning function), independent of language and culture, and can be performed within 2 h. Our analysis showed a clear association between clinical stage and reaction time, emphasizing the role of the number of chemotherapy cycles. Attention impairments were the most pronounced in our patients. Our investigation suggests that an older age and inactive employment status require enhanced attention. Their cognitive functions and, through that, their quality of life can be improved if they return to work or, if it is not possible, they may receive cognitive training. Our results draw attention to the fact that cognitive impairment is a real problem in patients with cured HL, similarly to other malignant diseases. Further longitudinal clinical studies could help characterize the neurocognitive outcomes of HL survivors.

## Supplementary Information

Below is the link to the electronic supplementary material.

Supplementary Table 1. The treatment protocols based on the stage of the disease A(E)BVD: adriamycin (epirubicin), bleomycin, vinblastine, and dacarbazine; CV(O)PP: cyclophosphamide, vinblastine [vincristine], procarbazine, and prednisolone; COPP/ABV: cyclophosphamide, vincristine, procarbazine, prednisolone/adriamycin, bleomycin, and vinblastine; Other protocols include MOPP: mustargen, vincristine, procarbazin, prednisolone, OEPA: vincristine, etoposide, prednisone, and doxorubicin (PNG 57 kb)

High resolution image (TIF 277 kb)

Supplementary Table 2. Associations between cognitive subtests results and patient/treatment-related factors among HL survivors (PNG 779 kb)

High resolution image (TIFF 1955 kb)

## Data Availability

Not applicable.
