# Supplementary figures and images for: Assessment of cognitive function in long-term Hodgkin lymphoma survivors, results based on data from a major treatment center in Hungary
Source: Support Care Cancer. 2022 Mar 11;30(6):5249–58. doi: 10.1007/s00520-022-06918-6 (PMC9046282; doi:10.1007/s00520-022-06918-6)

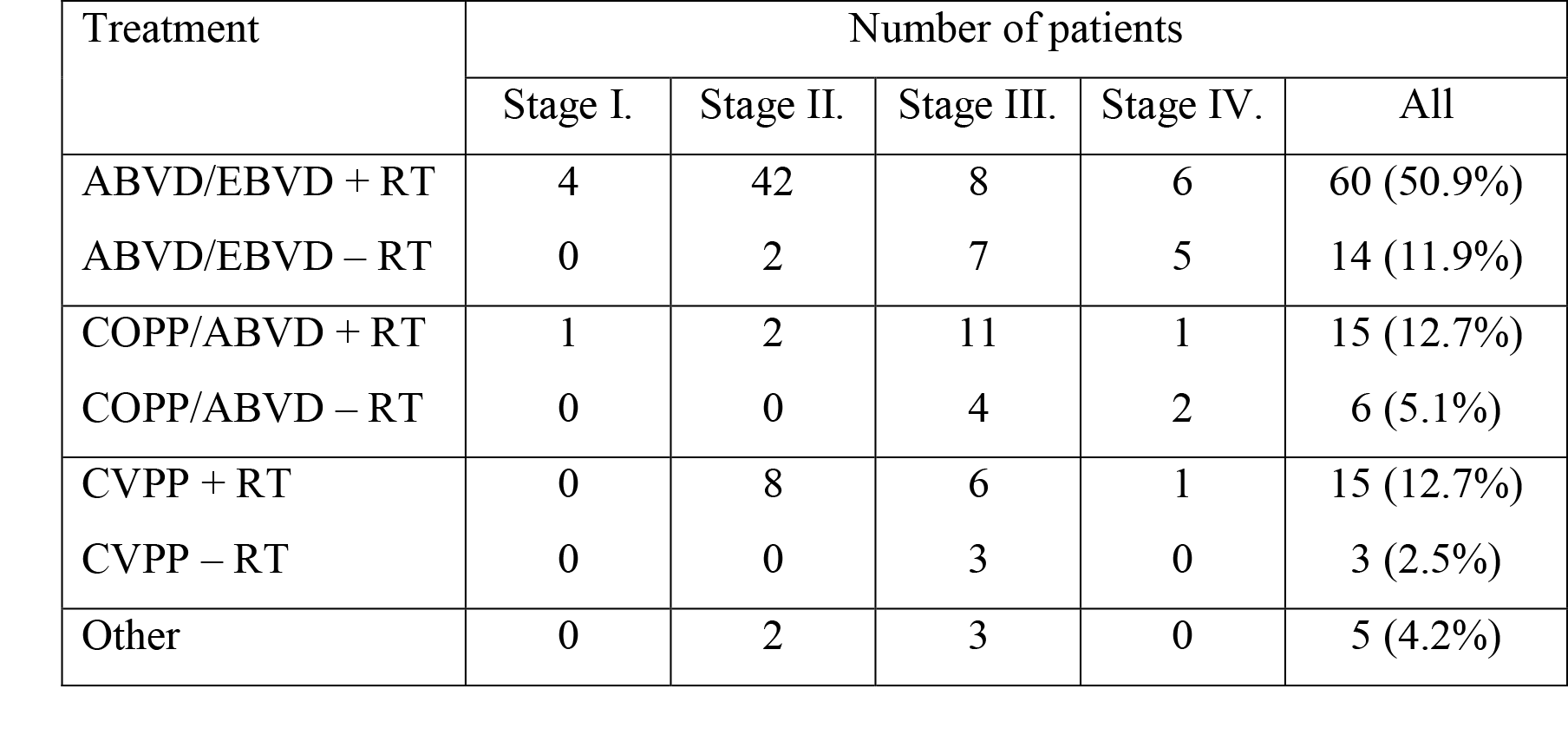

Supplement: Supplementary file 1 — The treatment protocols based on the stage of the disease A(E)BVD: adriamycin (epirubicin), bleomycin, vinblastine, and dacarbazine; CV(O)PP: cyclophosphamide, vinblastine [vincristine], procarbazine, and prednisolone; COPP/ABV: cyclophosphamide, vincristine, procarbazine, prednisolone/adriamycin, bleomycin, and vinblastine; Other protocols include MOPP: mustargen, vincristine, procarbazin, prednisolone, OEPA: vincristine, etoposide, prednisone, and doxorubicin (PNG 57 kb) [file 520_2022_6918_Fig3_ESM.png]

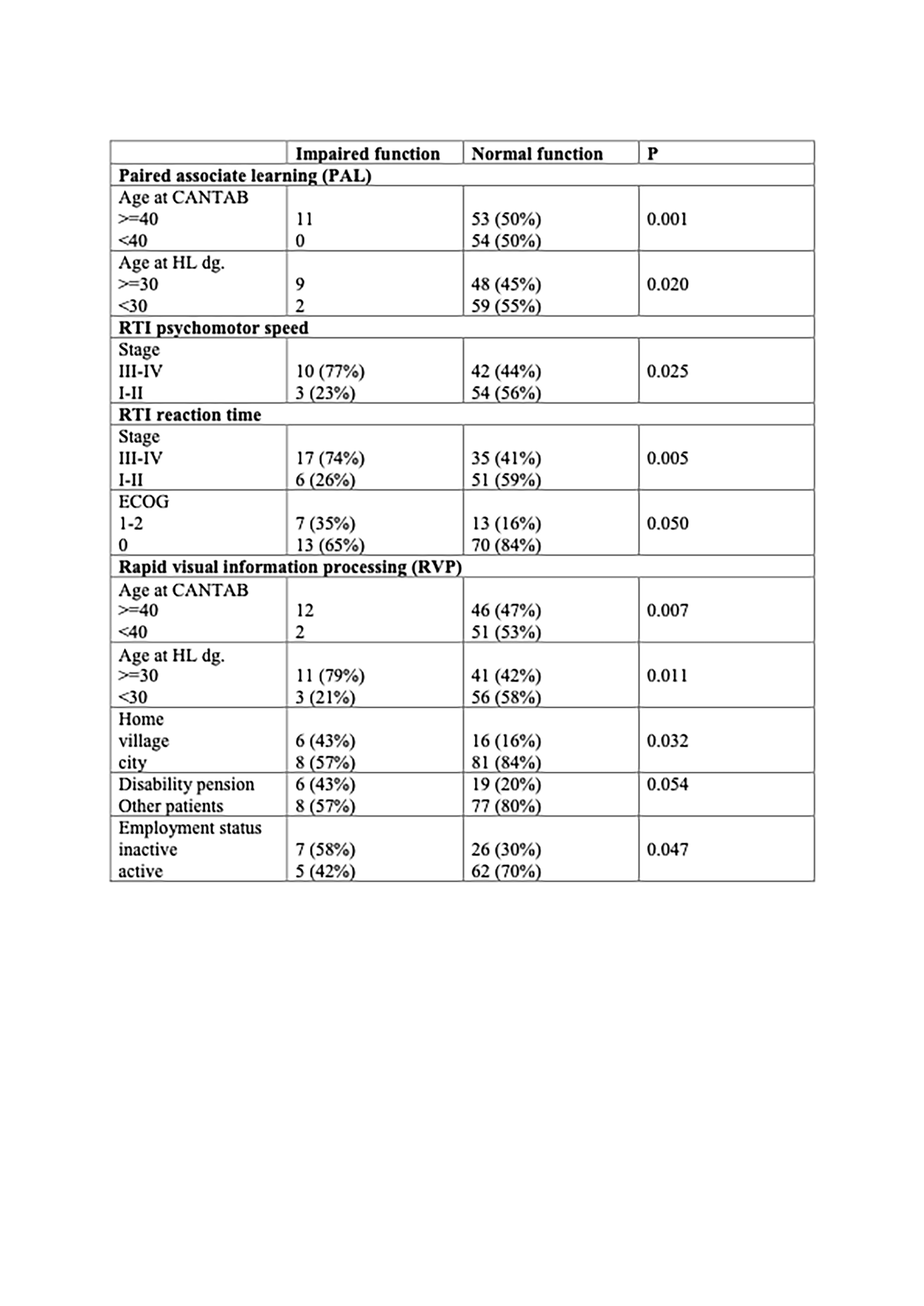

Supplement: Supplementary file 3 — Associations between cognitive subtests results and patient/treatment-related factors among HL survivors (PNG 779 kb) [file 520_2022_6918_Fig4_ESM.png]

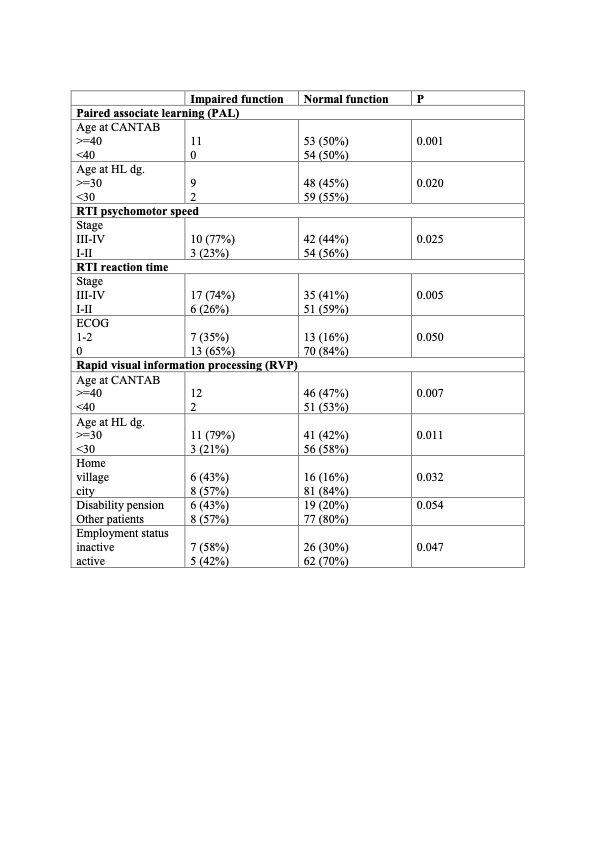

Supplement: Supplementary file 4 — High resolution image (TIFF 1955 kb) [file 520_2022_6918_MOESM2_ESM.tiff]
